# Comprehensive Phylogenetic Analysis of Bacterial Reverse Transcriptases

**DOI:** 10.1371/journal.pone.0114083

**Published:** 2014-11-25

**Authors:** Nicolás Toro, Rafael Nisa-Martínez

**Affiliations:** Grupo de Ecología Genética de la Rizosfera, Estación Experimental del Zaidín, Consejo Superior de Investigaciones Científicas (CSIC), Granada, Spain; University of Poitiers, France

## Abstract

Much less is known about reverse transcriptases (RTs) in prokaryotes than in eukaryotes, with most prokaryotic enzymes still uncharacterized. Two surveys involving BLAST searches for RT genes in prokaryotic genomes revealed the presence of large numbers of diverse, uncharacterized RTs and RT-like sequences. Here, using consistent annotation across all sequenced bacterial species from GenBank and other sources via RAST, available from the PATRIC (Pathogenic Resource Integration Center) platform, we have compiled the data for currently annotated reverse transcriptases from completely sequenced bacterial genomes. RT sequences are broadly distributed across bacterial phyla, but green sulfur bacteria and cyanobacteria have the highest levels of RT sequence diversity (≤85% identity) per genome. By contrast, phylum Actinobacteria, for which a large number of genomes have been sequenced, was found to have a low RT sequence diversity. Phylogenetic analyses revealed that bacterial RTs could be classified into 17 main groups: group II introns, retrons/retron-like RTs, diversity-generating retroelements (DGRs), Abi-like RTs, CRISPR-Cas-associated RTs, group II-like RTs (G2L), and 11 other groups of RTs of unknown function. Proteobacteria had the highest potential functional diversity, as they possessed most of the RT groups. Group II introns and DGRs were the most widely distributed RTs in bacterial phyla. Our results provide insights into bacterial RT phylogeny and the basis for an update of annotation systems based on sequence/domain homology.

## Introduction

Retrotransposons are mobile genetic elements that invade the genome and spread by the reverse transcription of an RNA transposition intermediate and the insertion of the resulting cDNA at a new site. The enzyme that converts the RNA into cDNA, reverse transcriptase (RT), was discovered in 1970 [1]–[2] in RNA tumor viruses (retroviruses). All retrotransposons have RTs, and these enzymes are classified into two main classes on the basis of their mechanism of transposition and DNA organization: 1) LTR-retrotransposons, which are characterized by direct repeats of a few hundred base pairs at their ends (long terminal repeats), generally containing the *gag* and *pol* genes; the Pol enzyme encoded by these sequences has several enzymatic domains (RT, RNase H, integrase, and PR or proteinase); and 2) non-LTR retrotransposons, which have no terminal repeats and are highly abundant in eukaryotes (known as LINEs in mammals). They contain a single ORF encoding a protein with RT and EN (endonuclease) domains or two ORFs, the first displaying similarity to the *gag* gene of retroviruses, and the second encoding the RT and EN domains. LTR retrotransposons are thought to have evolved from non-LTR retrotransposons through the acquisition of an integrase, and retroviruses are thought to have evolved from LTR retrotransposons through the incorporation of envelope (*env* domain) genes from other viruses [3], [4]. Retrotransposons have greatly influenced eukaryotic genomes, accounting for about 45% of the human genome and driving genome evolution and changes in gene expression [5].

Bacterial RTs were discovered much later, in a retroelement known as a retron [6]–[7], which synthesizes a large number of multicopy single-stranded DNA (msDNA) molecules. Retrons are thought to have no independent mobility, and their function remains unknown. Another type of bacterial retroelement, the DGR (diversity-generating retroelement), was first described in 2002 [8]. DGRs consist of an RT, an accessory protein (encoded by the *atd* gene), an RNA template, and a gene encoding the target protein (*mtd*), which contains a C-terminal variable region (VR). DGRs do not appear to be mobile, but they produce diverse sequences in the VR region, potentially conferring resistance against phages [9]–[10]. Bacteria have other mechanisms of antiphage immunity, including CRISPR (clustered regularly interspaced, short palindromic repeats) and Abi (abortive bacteriophage infection) systems. CRISPR and their *cas*-associated genes encode a sequence-specific mechanism of defense against bacteriophages and plasmids [11]–[13], consisting of an array of short repetitive sequences (∼40 bp long) separated by equally short spacer sequences. Two uncharacterized classes of bacterial RTs have been shown to be associated with CRISPR/cas elements, and some RTs are fused to *cas* genes [14]–[15], but their role in CRISPR function is unknown. The Abi system blocks phages at different stages in the infection cycle and is generally mediated by a single plasmid-encoded gene. The AbiA, AbiK, and Abi-P2 systems are known to include a RT [16]–[18]. Mutations of the gene encoding the RT of AbiK have been shown to block infection [16], and this enzyme has recently been shown to have genuine RT activity [19].

The best characterized mobile bacterial retroelements are the catalytic RNAs known as group II introns. These mobile elements were first identified in the mitochondrial and chloroplast genomes of lower eukaryotes and plants, and have subsequently been described in bacteria and archaea [20]–[24]. Group II introns consist of a structured RNA that folds into a conserved three-dimensional structure organized into six double-helical domains, DI to DVI [25]. Most bacterial group II introns have an open reading frame (ORF) encoding an intron-encoded protein (IEP) in DIV. This IEP consists of an RT followed by a putative RNA-binding domain with RNA splicing or maturase activity (the X domain), and, in some intron lineages, a C-terminal DNA-binding and endonuclease domain [26]–[28]. It is thought that both nuclear spliceosomal introns and non-LTR retrotransposons evolved from mobile group II introns [26]–[32], which may also influence bacterial evolution [33].

Much less is known about the RTs of prokaryotes than about those of eukaryotes, and many of these enzymes remain uncharacterized. Two surveys [14]–[15] investigating the presence of RT sequences in prokaryotic genomes revealed large numbers of diverse, uncharacterized RTs and RT-like sequences, which were grouped into 20 phylogenetic classes with 11 domain architectures [15]. Here, we used consistent annotation across all sequenced bacterial species from GenBank and other sources, via RAST [34], [35], available from the PATRIC (Pathogenic Resource Integration Center) platform [36], to compile data for the currently annotated reverse transcriptases from completely sequenced bacterial genomes. Phylogenetic analyses revealed that bacterial RTs could be classified into 17 main groups. We provide strong evidence for the definition of several classes of RT that could provide the basis for a more comprehensive annotation of RT sequences in databases.

## Materials and Methods

### Collation of the RT dataset

For phylogenetic analyses, we used an RT (RT 0–7 domains) dataset described in a previous study [28] and including representatives of group II (GII) intron ORFs of subclasses A, C, D, E (E1/E2), F and the new variety *g*1, with no recognizable D/En region (En^−^), together with representatives of subclasses B, CL (CL1A, CL1B, CL2A, and CL2B), and ML, with a recognizable D/En region (En^+^). The GII RT dataset included 153 unique sequences that were ≤85% identical, with the exception of the subclass A RT sequences E.c.I4 and S.f.I1, because the bacterial group II intron database (≠http://webapps2.ucalgary.ca/~groupii/index.html≠) contains only three reported members of this subclass, which are almost identical (99% identity). Other uncharacterized bacterial RT sequences [15] corresponding to the group II intron-like groups G2L1-5 and G2L-other (24), retrons/retron-like RTs (113), DGRs (36), the abortive phage infection AbiK (3) and Abi-P2 (8) groups, and other unknown RT groups (UG) 1–9 and unclassified (UN) elements (118) were then added to the group II intron RTs. Multiple sequence alignments (MSAs) including only sequences displaying ≤85% identity were generated for this dataset and subjected to various phylogenetic analyses, to determine the consistency of the monophyly previously proposed for the different groups [15], and to select reliable representative members. AbiA sequences and sequences for the group II intron-like G2L5 group were not included in the final dataset, because the AbiA sequences displayed poor conservation of the RT domain and the G2L5 sequences did not form a consistent supported group in our analyses. The final dataset (Table S1) contained 277 sequences, 124 of which were assigned to RT groups other than group II intron ORFs.

### Search for annotated bacterial RTs

We used the PATRIC platform to search for annotated bacterial RTs. This platform offers consistent annotation across all sequenced bacterial species from GenBank and other sources via RAST, the most widely used and highly cited automated microbial annotation system [34]–[36]. For practical purposes, the search was carried out independently for the various phyla and classes for which complete genome sequences were available. At the time of analysis (06/23/2014), the database contained 239 complete genomes for Alphaproteobacteria, 144 for Betaproteobacteria, 570 for Gammaproteobacteria and 156 for Delta/Epsilon Proteobacteria. It also contained 556 complete genomes from phylum Firmicutes, including 418 from bacilli and 138 from clostridia. Complete genome sequences were available for 339 actinobacteria, 98 members of the Bacteroidetes/green sulfur bacteria (phylum Chlorobi) group and 72 cyanobacteria. We searched the PATRIC database with the Feature Finder tool, searching for reverse transcriptase coding sequences (CDS). The product sequence did not refer exclusively to reverse transcriptases, but included heterogeneous annotations, such as retron-type RNA-directed DNA polymerase, retron-type reverse transcriptase, mobile element protein, hypothetical protein, conserved domain protein, RNA-directed DNA polymerase, probable group II intron maturase/reverse transcriptase, and ribonuclease III. Nevertheless, the keyword “reverse transcriptase” was present in the corresponding annotation in the PATRIC, BRC or Refseq database and a conserved RT domain was identified in all cases. Sequences were downloaded and analyzed with Geneious Pro software (Biomatters Ltd.), and proteins of less than 200 amino acids in length were discarded as probable fragments. We then used MAFFT and the BLOSUM62 scoring matrix to align sequences for each phylum. The % identity obtained with the distance matrix for the alignment was used to identify protein sequences displaying ≤85% identity overall. For further analyses, we retained 662 sequences: 293 sequences from Proteobacteria, 182 from Firmicutes, 22 from Actinobacteria, 60 from Bacteroidetes/green sulfur bacteria and 105 from Cyanobacteria. We used MAFFT to align these sequences with the 277 RT sequences of the initial dataset, and the amino-acid region corresponding to RT domains 0 to 7 was then extracted. The annotated RTs from PATRIC displaying ≤85% identity (sequences displaying >85% identity were not found across phyla) and different from the initial dataset (465 sequences) were retained: 209 from Proteobacteria, 128 from Firmicutes, 13 from Actinobacteria, 42 from Bacteroidetes/green sulfur bacteria and 73 from Cyanobacteria. We used MAFFT to generate a final alignment with 742 sequences (Figure S1), and 100 bootstrap repetitions were carried out with GUIDANCE (http://guidance.tau.ac.il) [37] to obtain alignment confidence scores for each pair of residues. The final alignment used for the phylogenetic analyses contained 254 informative positions.

### Phylogenetic analyses

Phylogenetic analysis was performed with several different maximum likelihood estimation methods implemented in Geneious Pro (Biomatters Ltd.) and Cipres Science Gateway platform [38]. We generated unrooted trees with FastTree [39]–[40], using pseudocounts (recommended for sequences containing large numbers of gaps), a discrete gamma model with 20 rate categories (“Gamma20”) for between-site rate variation, and the WAG model of amino-acid evolution. Local support values for branches were obtained by applying the Shimodaira-Hasegawa test to three alternative topologies (NNIs) around the split. It has been suggested that, for very large phylogenies, FastTree is less sensitive to alignment and dataset size than other maximum likelihood methods that estimate phylogeny very quickly with little or no loss of tree accuracy [40]. The other methods used included RAxML [41], with the LG amino-acid substitution model and a discrete gamma model with four categories and 400 bootstraps.

## Results and Discussion

### Insights from bacterial RT phylogeny

**Figure 1:**
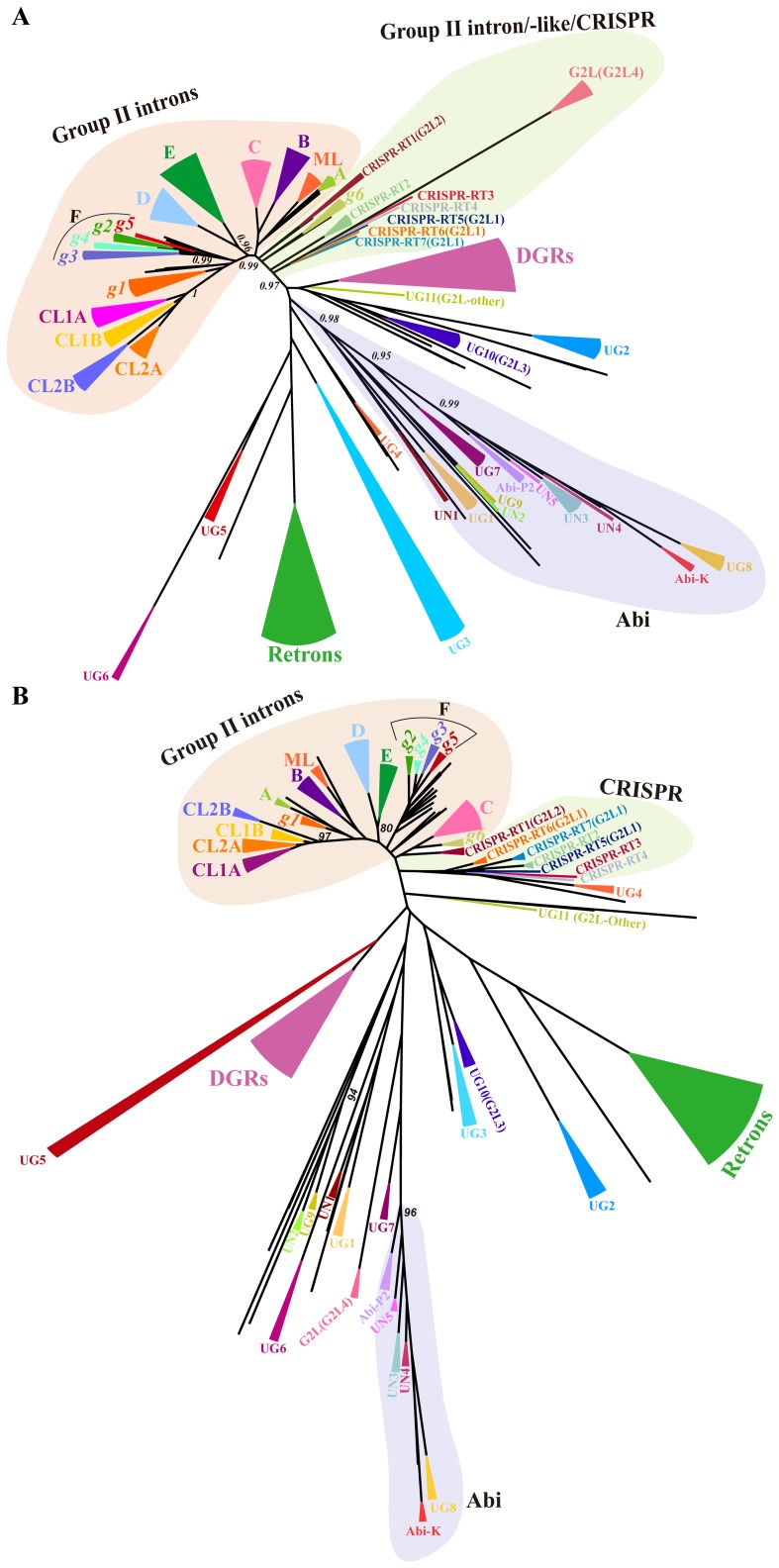
Bacterial RT phylogeny. Unrooted phylogenetic trees based on the alignment of the RT-domain (domains 0–7) of the 742 sequences used as the dataset. The group II intron ORF classes and varieties are designated according to the most recent proposals from Toro *et al.* in 2013 and Simon *et al.* in 2008 [28], [42]. Additional group II intron ORF varieties are also annotated here (*g5* and *g6*, see the results and discussion section). Other RT groups are named as previously reported [15] and suggested in this work. (*A*) A consensus unrooted tree estimated by FastTree method. The main groups were color-coded to improve visualization and collapsed at the internal nodes with local support values ≧0.96, with the exceptions of UG4 (0.84) and UG5 (0.87). Local support values ≧0.95 at inner nodes are also indicated. (*B*) A consensus unrooted tree estimated by the RAxML method. The main groups were collapsed at the internal nodes with strong support (bootstrap values ≧75%), with the exception of class ML (55%), subgroup CL1B (34%) and variety *g3* (71%); UG10 (G2L3) (39%); and DGRs (17%). Inner nodes with strong statistical support (bootstrap values ≧80%) are also indicated.

Annotated RTs were retrieved from the PATRIC database, as indicated in the Materials and Methods. Searches were performed for most bacterial phyla, but only RT sequences from phyla for which larger complete sequenced genomes were available (Proteobacteria, Firmicutes, Actinobacteria, the Bacteroidetes/green sulfur bacteria group and Cyanobacteria) and with large numbers of annotated RTs were included in the phylogenetic analyses. We generated a final dataset containing 742 RT sequences, and a MSA was constructed by GUIDANCE (Figure S1), for the generation of unrooted phylogenetic trees with FastTree (Figure 1A) and RAxML (Figure 1B).

#### Group II intron ORFs

The FastTree phylogeny (Figure 1A) strongly supported all previously described GII ORF classes, A, B, C, D, E, CL, ML, and the new varieties *g1* to *g4* with a local value of 0.96 to 1. The RAxML phylogeny (Figure 1B) also supported the ORF classes and varieties (bootstrap values of 71% to 100%), but provided low levels of statistical support for class ML and subgroup CL1B. The RAxML method may be more sensitive to the alignment and dataset size, due to the small number of characters (254 aa) in the alignment. Surprisingly, FastTree provided strong support (local value 0.99) for class F, for which weak statistical support was provided by the tree generated by RAxML (Figure 1B), and by other previous maximum likelihood estimations [28], [42]. Class F corresponds to the previously described varieties *g2–g4* [28], including c-Ku.st.I1, and the So.us.I2, Ge.ur.I1 and Pe.th.I2 RT sequences that cluster together in the inferred trees (this cluster is referred to below as variety *g5*), for which there was a high degree of support (local value of 0.96 and bootstrap value of 90%). Classes D and E appear to be sister clades in both trees (local value of 0.96 and bootstrap value of 80%), consistent with the findings of a previous study [28]. Some RTs from class C introns (D.pI1, Pe.th.I1 and Al.or.I2) were previously shown to form a divergent subgroup [28]. In the phylogenetic trees generated by FastTree and RAxML, these RT sequences, together with sequences from other clostridia and gammaproteobacteria, including sequences from the current group II intron database (Na.th.I1, Tc.po.I1 and Al.ma.I1), clustered together (local value of 1 and bootstrap value of 92%) outside monophyletic class C, consistent with the monophyly of this subgroup (referred to hereafter as *g6*). The class C and *g6* introns have similar intron RNA structures [43], due to either convergent evolution under selective pressure or the reshuffling of intron RNAs and IEPs.

We previously suggested [28] that intron ORF En^+^ classes (B, ML, and CL) correspond to a monophyletic intron lineage. However, the FastTree and RAxML phylogenies showed that classes B and ML were separated from the CL class by several ORF En^−^ classes. Furthermore, in the FastTree phylogeny, classes CL, F, and variety *g1* branched from a strongly supported node (local value 0.99). However, neither FastTree with a shorter alignment (less aligned residues, not shown) nor RAxML yielded this tree topology. In the inferred tree with RAxML, class F was found outside of the ORF En^+^ nodes, and the inner common node for classes CL, A and the *g1* variety lacked statistical support. The inner nodes of the trees remain to be resolved, so relationships between En^+^ and En^−^ classes are less reliable with the large dataset including RTs other than group II intron ORFs.

#### RTs associated with CRISPR-Cas systems

RTs associated with CRISPR-Cas systems without recognizable intron RNA structures are located at the base of the group II intron lineage, together with the *g6* intron variant and other unclassified group II intron RTs. The current classification [44] includes three major types of CRISPR–Cas system (I, II and III), with a further division into several subtypes and some chimeric variants. None of the CRISPR-Cas systems with associated RTs belong to the type II lineage; some are classified as type III-A, but most are currently unclassified systems (type III subtype U), probably corresponding to CRISPR-Cas chimera. Some of these CRISPR-Cas-associated RTs, which have no maturase domain, form monophyletic groups with strong support (local support value of 1 and bootstrap value ≧98%). These groups include the G2L2 group (hereafter referred to as CRISPR-RT1), all four members of which are from green sulfur bacteria, and the CRISPR-RT2 group, which includes three RTs from cyanobacteria. The previously proposed G2L1 group [15], which is also associated with CRISPR-Cas elements, could be split into three distinct groups. Two groups potentially resulting from convergent evolution were characterized on the basis of their RTs being fused to Cas1-ORF proteins. One of these groups (hereafter referred to as CRISPR-RT6, with a local support value of 1 and a bootstrap value of 100%) comprised the green sulfur bacterial RTs from *Chlorobium phaeobacteroides* DSM 266 (gi|119357846) and *Pelodictyon phaeoclathratiforme* BU-1 (gi|68548733), and the second (hereafter referred to as CRISPR-RT5) corresponded to a single RT from the gammaproteobacterium *Vibrio vulnificus* YJ016 (gi|37677204). A third group was characterized by RTs consisting exclusively of the RT domain. This group included the RTs from *Candidatus kuenenia* (gi|91201518) and a RT from *Delsufobacca acetooxidans* (gi|328952997) (hereafter referred to as CRISPR-RT7, local support value of 0.98 and bootstrap value of 94%). Similarly, the phylogenetic tree revealed the existence of other RTs also associated with CRISPR-Cas systems, corresponding to a RT from *Thioflavococcus mobilis* (gi|431828084) and a RT from *Rhodomicrobium vannielii* (gi|312114615), hereafter referred to as CRISPR-RT3 and CRISPR-RT4, respectively. All the RTs associated with CRISPR-Cas systems were organized into seven conserved domains and the characteristic aspartate (DD) residues of the YADD motif located in the active site of the protein in domain 5 were 100% conserved. The tyrosine (Y) residue was replaced with a phenylalanine (F) residue in 66% of the sequences, whereas the alanine (A) residue in the second position was conserved in 66% of cases. These RTs may, therefore, have polymerase activity.

#### Group II-like intron ORFs with conserved maturase domains

**Figure 2:**
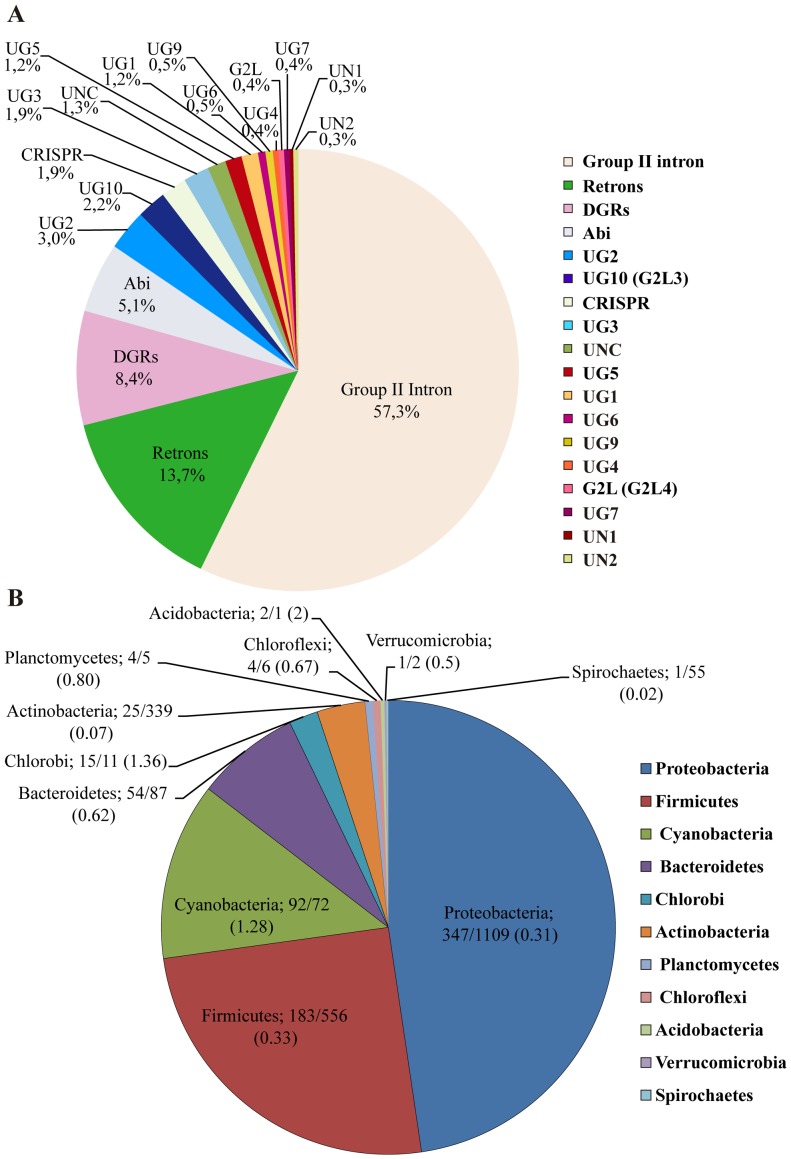
Bacterial RTs of the dataset in bacterial phyla. (*A*) Percentage of each of the RT groups in the dataset. Note that the Abi lineage includes here only those groups supported by FastTree and RAxML (AbiK; Abi-P2; UN3–5 and UG8). UNC are unclassified RTs. (*B*) RT sequences in the dataset (≤85% identity), by phylum. The numbers of RT sequences and currently completed genome sequences per phylum are indicated, together with their ratio.

Simon and Zimmerly [15] defined five group II-like groups (G2L1–5) and a group with a single sequence (G2L-other) as paraphyletic characterized by an apparent lack of intron RNA structure and a conserved maturase domain in the groups G2L3–5 and G2L-other. The G2L5 sequences (gi|149173121 and gi|149176144 from *Planctomyces maris* and gi|87308561 from *Blastopirullela marina*) did not form a consistent supported group in our preliminary phylogenetic analyses and they were not included in the initial dataset of 277 sequences. Nevertheless, the inclusion of these sequences in the final alignment and FastTree analysis revealed that the sequences from *Planctomyces* grouped together, but their relationships with the other RT sequences and defined RT groups remain uncertain, since the nodes lack statistical support and closed relatives to these sequences were not identified in our analysis (not shown). In our phylogenetic analyses with FastTree, G2L4 (2 sequences in the initial dataset), and a RT from *Methylocella silvestris* BL2 (gi| 217978298) formed a monophyletic group (local value of 1) that appeared to be interleaved with the CRISPR-associated RTs (Figure 1A), including the G2L2 (CRISPR-RT1) and G2L1 (CRISPR-RT5–7) groups. However, in the tree generated by RAxML (Figure 2B), G2L4 (bootstrap value of 100%) appeared at the base of a potential Abi lineage. G2L4 had a large number of substitutions per site (FastTree: 3.004 and RAxML: 2.3), suggesting that this group is evolving rapidly. It is therefore possible that the position of G2L4 in the phylogenetic analyses may be influenced by systematic errors caused by the mutational saturation of the G2L4 sequences. The three members of G2L4 contain the YIDD sequence rather than the canonical YADD motif in domain 5, and this is a distinctive unique feature of this group of RTs.

The single G2L-other sequence and the monophyletic G2L3 group (local value 0.96) were clearly separated from the other G2L groups by FastTree and RAxML methods. The G2L3 group is one of the larger RT groups, with 16 members from our dataset, and, in all these sequences, the alanine residue of the YADD motif is replaced with a valine (V) residue. Thus, despite their conserved maturase domain, G2L3 and G2L-other RTs may have an origin different from that of other G2L groups. We therefore suggest that these two groups of RTs should be renamed, with the nomenclature used for RTs of unknown function, hereafter referred to as UG10 (G2L-3) and UG11 (G2L-other), and that the G2L nomenclature should be retained only for the G2L4 group.

#### Retrons/retron-like RTs

A retron lineage is strongly supported in the phylogenies obtained with FastTree (local support value of 1) and RAxML (bootstrap value of 94%). In our analysis, this group expanded from six sequences in the initial dataset to 102 sequences with ≤85% identity. This constitutes a very large increase. The retron-specific conserved motif (VTG) within RT domain 7 in the “Y” region crucial for specific recognition and binding to the template-primer RNA used for the synthesis of msDNA [45] was present in 47% of the sequences, with variants including sequences with isoleucine/leucine (I/L) residue (26% of the sequences) in the first position. Moreover, the conserved NAXXH amino-acid motif in region “X”, located between domains 2 and 3, could be identified in 59% of the sequences. The histidine (H) residue displayed 100% conservation in these sequences, whereas the alanine (A) and asparagine (N) residues were conserved in 87% and 62% of cases, respectively. We did not attempt to identify potential msRNA/msDNA secondary structures, but our results suggest that the members of this cluster are actual retron RT sequences.

#### The Abi lineage

The phylogenetic analysis carried out with FastTree provided strong local support (0.98) for an Abi-related lineage of RTs, including the strongly supported groups (local values of 0.95 to 1) Abi K; Abi-P2; UN1-5 and UG1, 7–9. RAxML also supported a potential Abi-like lineage, but including only AbiK, Abi-P2, and the UN3–5 and UG8 groups. The relationships between these RT groups and the UN1–2, UG1, UG7 and UG9 groups therefore remain unclear. An analysis of the corresponding sequences of the Abi lineage (RAxML) showed them to have a mean length of 562 aa, with a C-terminal region of unknown domain architecture. These RTs lack the RT 0, 2a and 7 domains and the potentially active site in domain 5 has a conserved Y(R/V)DD sequence. It would therefore be of interest to determine whether these proteins share biochemical properties, and whether they are all engaged in the abortive infection pathway.

#### DGR RTs

In our analyses, a potential DGR clade containing 62 members was supported by the FastTree phylogeny (local support value of 0.94). The mean length of 375 aa for these sequences is consistent with the 378 aa reported as the mean length of DGR RTs [46]. It has been suggested that DGR RTs, although highly divergent, form a phylogenetic clade that is characterized by a (I/V/L)GxxxSQ motif in RT domain 4 [46], which is sufficient to predict DGR association. We found that 57 of the analyzed sequences contained the SQ motif in domain 4 and a Y(V/M)DD sequence in the active site of domain 5, which is also a feature of DGR RT sequences. The SQ residues varied in only five sequences, in which they were replaced by AQ or AN dipetides, or lost altogether, these variants probably corresponding to inactivation events or sequencing errors. The RAxML phylogeny did not provide strong support for this clade (instead it strongly supported 10 outer nodes within the clade grouping 46 sequences), but the FastTree phylogeny and the presence of the SQ motif suggest that the grouped sequences are DGR/DGR-like RTs.

### Phylogenetic distribution of RT groups

**Figure 3:**
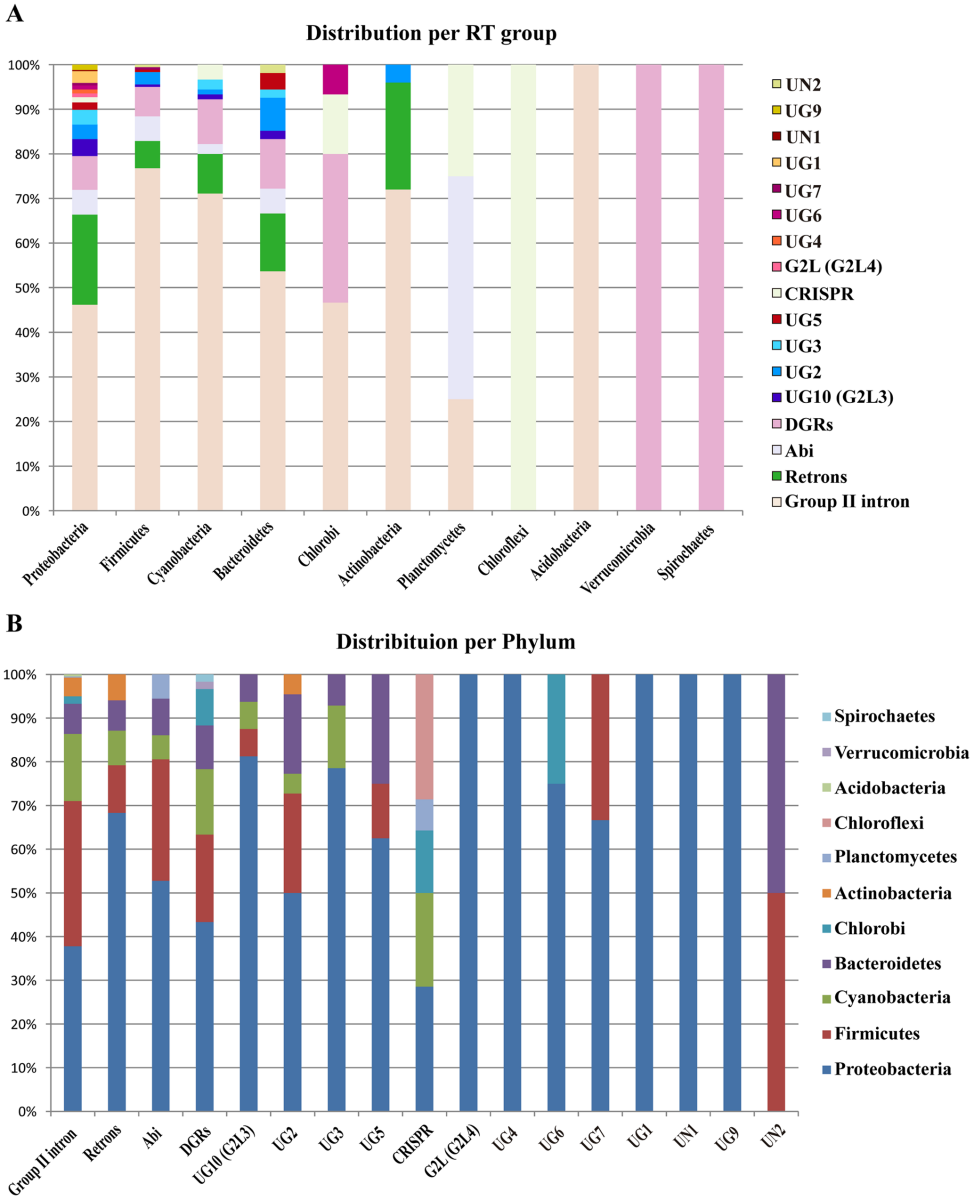
Phylogenetic distribution of RT sequences in bacteria. (*A*) Distribution per phylum. (*B*) Distribution per RT class. Sector colors represent the percentage of the corresponding RT group or phylum.

About 57% of the bacterial RTs in the dataset corresponded to group II intron-encoded ORFs. The next most frequent groups were retrons (13.7%), DGRs (8.4%) and RTs of the Abi lineage (5.1%). However, the functions of bacterial RT sequences appear to be highly diverse, as many others could be classified into different minority groups of unknown function (Figure 2A). RT sequences were found to be broadly distributed across bacterial phyla, the number of RT sequences being generally correlated with the number of genomes sequenced, but, as shown in Figure 2B, the normalized sequence diversity (ratio of RTs ≤85% identity per genome) was higher for green sulfur bacteria (1.36) and Cyanobacteria (1.28), the values obtained for Proteobacteria and Firmicutes being much lower, at only 0.31 and 0.33, respectively. By contrast, phylum Actinobacteria, for which a large number of genomes have been sequenced (339), had a low diversity of RT sequences (0.07) (Figure 2B). If we consider the distribution per RT class or per phylum (Figure 3A and 3B), Proteobacteria display the greatest functional diversity, containing most groups, with the exception of UN2, which appears to be restricted to Bacteroidetes and Firmicutes. Moreover, five groups (G2L4, UG4, UG1, UN1 and UG9) are restricted to Proteobacteria. Group II introns and DGRs appear to be the RTs most widely distributed in bacterial phyla, followed by retrons, Abi, UG2 and CRISPR-Cas RTs. These RT groups appear to be the most relevant in bacteria, but minority groups may correspond to evolving RTs with novel properties and functions.

## Conclusions

We provide here an overview of current knowledge about bacterial group II intron RTs and the groups previously proposed on the basis of RT annotation with the PATRIC platform, which provides consistent annotation across all sequenced bacterial species from GenBank and other sources, via RAST. The application of the widely recognized RAST procedure to all available genomes made it possible to detect RT sequences in an unbiased manner, based on the most up-to-date data, rather than making use of potentially incomplete Blast searches, or missing or incorrect annotations. Several different ML estimation methods were used for the phylogenetic analyses and FastTree was found to estimate phylogeny with little or no loss of accuracy for the clustering of major RT groups, with lower sensitivity to dataset size than the RAxML method. However, the relationships between major classes remain unresolved and inconsistencies were observed between the results obtained with the FastTree and RAxML methods. We found that bacterial RTs could be classified into 17 main groups: group II introns, retrons/retron-like RTs, diversity-generating retroelements (DGRs), Abi-like RTs, CRISPR-Cas associated RTs, group II-like RTs (G2L), and 11 other groups of RTs of unknown function. We found that RT sequence diversity was greatest in green sulfur bacteria and cyanobacteria, but that proteobacteria displayed potentially greater functional diversity, with some groups restricted to this phylum. Group II introns and DGRs were the most widely distributed RTs in bacterial phyla. Our results provide a basis for future studies of RT properties and function, together with new insight into bacterial RT phylogeny, which may facilitate an updating of annotation systems based on sequence/domain homology.

## Supporting Information

Figure S1 **MSA alignment of the 742 RT sequences (domains 0–7) used to construct the phylogenetic trees shown in** **Figure 1** **.** The accession and identification numbers provided by the PATRIC (fid) and GenBank (gi) databases are shown, together with phylum information. For Proteobacteria, an A (Alfa), B (Beta), G (Gamma) or DE (Delta/Epsilon) is used to distinguish between classes.
(FASTA)

Table S1 **The RT sequences used in the phylogenetic analysis shown in** **Figure 1** **are indicated, together with their main features, phylum, species, accession numbers and domain annotations, as they appear in the GenBank database.** The shadowed sequences correspond to the initial data set of 277 sequences. The names of the group II intron ORFs currently present in the group II intron database or as previously published [28] are indicated, together with information about whether the sequences were previously reported (Y) by Simon *et al.* [15]. The asterisk in the Abi classification indicates the sequences from the FastTree phylogeny lying outside the Abi lineage according to the RAxML estimation method.
(XLSX)

## References

[1] Baltimore D (1970) RNA-dependent DNA polymerase in virions of RNA tumour viruses. Nature 226 (5252):1209–1211 10.1038/2261209a0 4316300

[2] Temin HM, Mizutani S (1970) RNA-dependent DNA polymerase in virions of Rous sarcoma virus. Nature 226 (5252):1211–1213 10.1038/2261211a0 4316301

[3] Eickbush TH, Jamburuthugoda VK (2008) The diversity of retrotransposons and the properties of their reverse transcriptases. Virus Res 134(1–2):221–234 10.1016/j.virusres.2007.12.010 18261821 PMC2695964

[4] Finnegan DJ (2012) Retrotransposons. Current Biology 22 (11):432–437 10.1016/j.cub.2012.04.025 22677280

[5] Burns KH, Boeke JD (2012) Human transposon tectonics. Cell 149:740–752. 10.1016/j.cell.2012.04.019 22579280 PMC3370394

[6] Lampson BC, Inouye M, Inouye S (1989) Reverse transcriptase with concomitant ribonuclease H activity in the cell-free synthesis of branched RNA-linked msDNA of *Myxococcus xanthus* . Cell 56(4):701–707. 2465091 10.1016/0092-8674(89)90592-8

[7] Lim D, Maas WK (1989) Reverse transcriptase-dependent synthesis of a covalently linked, branched DNA-RNA compound in *E. coli B*. Cell. 56(5):891–904. 10.1016/0092-8674(89)90693-4 2466573

[8] Liu M, Deora R, Doulatov SR, Gingery M, Eiserling FA, et al (2002) Reverse transcriptase-mediated tropism switching in *Bordetella* bacteriophage. Science 295:2091–2094. 11896279 10.1126/science.1067467

[9] Medhekar B, Miller JF (2007) Diversity-generating retroelements. Curr Opin Microbiol 10(4):388–395. 17703991 10.1016/j.mib.2007.06.004 PMC2703298

[10] Bikard D, Marraffini LA (2012) Innate and adaptive immunity in bacteria: mechanisms of programmed genetic variation to fight bacteriophages. Curr Opin Immunol 24:15–20 10.1016/j.coi.2011.10.005 22079134

[11] Deveau H, Garneau JE, Moineau S (2010) CRISPR/Cas system and its role in phage-bacteria interactions. Annu Rev Microbiol 64:475–93 10.1146/annurev.micro.112408.134123 20528693

[12] Horvath P, Barrangou R (2010) CRISPR/Cas, the immune system of bacteria and archaea. Science 327(5962):167–70 10.1126/science.1179555 20056882

[13] Marraffini LA, Sontheimer EJ (2010) CRISPR interference: RNA-directed adaptive immunity in bacteria and archaea. Nat Rev Genet 11:181–190 10.1038/nrg2749 20125085 PMC2928866

[14] Kojima KK, Kanehisa M (2008) Systematic survey for novel types of prokaryotic retroelements based on gene neighborhood and protein architecture. Mol Biol Evol 25:1395–1404. 18391066 10.1093/molbev/msn081

[15] Simon D, Zimmerly S (2008) A diversity of uncharacterized retroelements in bacteria. Nucleic Acids Res 36:7219–7229. 19004871 10.1093/nar/gkn867 PMC2602772

[16] Fortier LC, Bouchard JD, Moineau S (2005) Expression and site-directed mutagenesis of the lactococcal abortive phage infection protein AbiK. J Bacteriol 187:3721–3730. 15901696 10.1128/JB.187.11.3721-3730.2005 PMC1112063

[17] Durmaz E, Klaenhammer TR (2007) Abortive phage resistance mechanism AbiZ speeds the lysis clock to cause premature lysis of phage-infected *Lactococcus lactis* . J Bacteriol 189:1417–1425. 17012400 10.1128/JB.00904-06 PMC1797342

[18] Odegrip R, Nilsson AS, Haggard-Ljungquist E (2006) Identification of a gene encoding a functional reverse transcriptase within a highly variable locus in the P2-like coliphages. J Bacteriol 188:1643–1647. 16452449 10.1128/JB.188.4.1643-1647.2006 PMC1367236

[19] Wang C1, Villion M, Semper C, Coros C, Moineau S, *et al* (2011) A reverse transcriptase-related protein mediates phage resistance and polymerizes untemplated DNA *in vitro* . Nucleic Acids Res 39(17):7620–7629 10.1093/nar/gkr397 21676997 PMC3177184

[20] Michel F, Ferat JL (1995) Structure and activities of group II introns. Annu Rev Biochem 64:435–461. 7574489 10.1146/annurev.bi.64.070195.002251

[21] Dai L, Zimmerly S (2003) ORF-less and RT-encoding group II introns in archaebacteria, with a pattern of homing into related group II intron ORFs. RNA 9:14–19. 12554871 10.1261/rna.2126203 PMC1370365

[22] Toro N (2003) Bacteria and Archaea group II introns; additional mobile genetic elements in the environment. Environ Microbiol 5:143–151. 12588294 10.1046/j.1462-2920.2003.00398.x

[23] Lambowitz AM, Zimmerly S (2004) Mobile group II introns. Annu Rev Genet 38:1–35. 15568970 10.1146/annurev.genet.38.072902.091600

[24] Toro N, Jiménez-Zurdo JI, García-Rodríguez FM (2007) Bacterial group II introns: not just splicing. FEMS Microbiol Rev 31:342–58. 17374133 10.1111/j.1574-6976.2007.00068.x

[25] Michel F, Costa M, Westhof E (2009) The ribozyme core of group II introns: a structure in want of partners. Trends Biochem Sci 34:189–99. 19299141 10.1016/j.tibs.2008.12.007

[26] Mohr G, Perlman PS, Lambowitz AM (1993) Evolutionary relationships among group II intron-encoded proteins and identification of a conserved domain that may be related to maturase function. Nucleic Acids Res 21:4991–4997. 8255751 10.1093/nar/21.22.4991 PMC310608

[27] San Filippo J, Lambowitz AM (2002) Characterization of the C-terminal DNA-binding/DNA endonuclease region of a group II intron-encoded protein. J Mol Biol 324:933–951. 12470950 10.1016/s0022-2836(02)01147-6

[28] Toro N, Martínez-Abarca F (2013) Comprehensive phylogenetic analysis of bacterial group II intron-encoded ORFs lacking the DNA endonuclease domain reveals new varieties. PLoS One 8(1):e55102 10.1371/journal.pone.0055102 23355907 PMC3552965

[29] Sharp PA (1985) On the origin of RNA splicing and introns. Cell 42:397–400. 2411416 10.1016/0092-8674(85)90092-3

[30] Cech TR (1986) The generality of self-splicing RNA: relationship to nuclear mRNA splicing. Cell 44:207–210. 2417724 10.1016/0092-8674(86)90751-8

[31] Cavalier-Smith T (1991) Intron phylogeny: a new hypothesis. Trends Genet 7:145–148. 2068786

[32] Eickbush TH (1994) Origins and evolutionary relationships of retroelements. In: Morse SSeditor. The Evolutionary Biology of Viruses. New York, NY: Raven Press, Inc. p. 121–157.

[33] Toro N, Martínez-Rodríguez L, Martínez-Abarca F (2014) Insights into the history of a bacterial group II intron remnant from the genomes of the nitrogen-fixing symbionts *Sinorhizobium meliloti* and *Sinorhizobium medicae* . Heredity 10.1038/hdy.2014.32 PMC4181065 24736785

[34] Aziz RK, Bartels D, Best AA, DeJongh M, Disz T, et al (2008) The RAST Server: rapid annotations using subsystem technology. BMC Genomics 9:75 10.1186/1471-2164-9 18261238 PMC2265698

[35] Overbeek R, Olson R, Pusch GD, Olsen GJ, Davis JJ, et al (2014) The SEED and the Rapid Annotation of microbial genomes using Subsystems Technology (RAST). Nucl Acids Res 42 (D1):D206–D214 10.1093/nar/gkt1226 PMC3965101 24293654

[36] Wattam AR, Abraham D, Dalay O, Disz TL, Driscoll T, et al (2014) PATRIC, the bacterial bioinformatics database and analysis resource. Nucleic Acids Res 42 (D1):D581–D591 10.1093/nar/gkt1099 PMC3965095 24225323

[37] Penn O, Privman E, Ashkenazy H, Landan G, Graur D, et al (2010) GUIDANCE: a web server for assessing alignment confidence scores. Nucleic Acids Res 38(suppl):W23–W28. 20497997 10.1093/nar/gkq443 PMC2896199

[38] Miller MA, Pfeiffer W, and Schwartz T (2010) Creating the CIPRES Science Gateway for inference of large phylogenetic trees” in Proceedings of the Gateway Computing Environments Workshop (GCE), 14 Nov. 2010, New Orleans, LA pp 1–8.

[39] Price MN, Dehal PS, Arkin AP (2009) FastTree: computing large minimum evolution trees with profiles instead of a distance matrix. Mol Biol Evol 26:1641–1650. 19377059 10.1093/molbev/msp077 PMC2693737

[40] Price MN, Dehal PS, Arkin AP (2010) FastTree 2 – approximately maximum-likelihood trees for large alignments. PLoS One 5, e9490. doi:10.1371/journal.pone.0009490. 10.1371/journal.pone.0009490 PMC2835736 20224823

[41] Stamatakis A (2006) RAxML-VI-HPC: maximum likelihood-based phylogenetic analyses with thousands of taxa and mixed models. Bioinformatics 22:2688–2690. 16928733 10.1093/bioinformatics/btl446

[42] Simon DM, Clarke NAC, McNeil BA, Johnson I, Pantuso D, et al (2008) Group II introns in Eubacteria and Archaea: ORF-less introns and new varieties. RNA 14(9):1704–1713. 18676618 10.1261/rna.1056108 PMC2525955

[43] Candales MA, Duong A, Hood KS, Li T, Neufeld RAE, et al (2011) Database for bacterial group II introns. Nucleic Acids Res. 40:187–190. 10.1093/nar/gkr1043 PMC3245105 22080509

[44] Makarova KS, Haft DH, Barrangou R, Brouns SJ, Charpentier E, et al (2011) Evolution and classification of the CRISPR-Cas systems. Nat Rev Microbiol 9:467–477. 21552286 10.1038/nrmicro2577 PMC3380444

[45] Inouye S, Hsu M-Y, Xu A, Inouye M (1999) Highly specific recognition of primer RNA structures for 2)-OH priming reaction by bacterial reverse transcriptases. J Biol Chem 274:31236–31244. 10531319 10.1074/jbc.274.44.31236

[46] Schillinger T, Lisfi M, Chi J, Cullum J, Zingler N (2012) Analysis of a comprehensive dataset of diversity retroelements generated by the program DIGReF. BMC Genomics 13:430–444. 22928525 10.1186/1471-2164-13-430 PMC3521204

